# Potential beneficial effects of a gluten-free diet in newly diagnosed children with type 1 diabetes: a pilot study

**DOI:** 10.1186/s40064-016-2641-3

**Published:** 2016-07-07

**Authors:** Jannet Svensson, Stine Møller Sildorf, Christian B. Pipper, Julie N. Kyvsgaard, Julie Bøjstrup, Flemming M. Pociot, Henrik B. Mortensen, Karsten Buschard

**Affiliations:** Copenhagen Diabetes Research Center (CPH-DIRECT), Department of Children and Adolescents, Copenhagen University Hospital Herlev, Herlev Ringvej 75, 2730 Herlev, Denmark; Section of Biostatistics, Department of Public Health, Faculty of Health and Medical Sciences, University of Copenhagen, Øster Farimagsgade, 1014 Copenhagen, Denmark; The Bartholin Institute, Rigshospitalet, Copenhagen Biocenter, Ole Maaløes Vej 5, 2200 Copenhagen N, Denmark

**Keywords:** Type 1 diabetes, Remission phase, Gluten, Insulin dose-adjusted HbA1c

## Abstract

**Aim:**

Gluten-free diet has shown promising effects in preventing type 1 diabetes (T1D) in animals as well as beneficial effects on the immune system. Gluten-free diet at diabetes onset may alter the natural course and outcome of autoimmune diseases such as T1D.

**Methods:**

In a 12-month study, 15 children newly diagnosed with T1D were instructed to follow a gluten-free diet. Questionnaires were used to evaluate adherence to the gluten-free diet. Partial remission (PR) was defined by insulin dose-adjusted A1c (IDAA1c) ≤9 or stimulated C-peptide (SCP) >300 pmol/L measured 90 min after a liquid mixed meal at the inclusion, six and 12 months after onset. The intervention group was compared with two previous cohorts. Linear mixed models were used to estimate differences between cohorts.

**Results:**

After 6 months, more children on a gluten-free diet tended to have SCP values above 300 pmol/L compared to the European cohort (p = 0.08). The adherence to a gluten-free diet decreased during the 12-month study period. After 1 year there was no difference in SCP levels or percentage in remission according to SCP (p > 0.1). Three times as many children were still in PR based on IDAA1c (p < 0.05). Twelve months after onset HbA1c were 21 % lower and IDAA1c >1 unit lower in the cohort on a gluten-free diet compared to the two previous cohorts (p < 0.001).

**Conclusion:**

Gluten-free diet is feasible in highly motivated families and is associated with a significantly better outcome as assessed by HbA1c and IDAA1c. This finding needs confirmation in a randomized trial including screening for quality of life. (Clinicaltrials.gov number NCT02284815).

## Background

Maintained endogenous insulin production in patients with type 1 diabetes (T1D) is associated with better metabolic control and a lower prevalence of adverse events, including hypoglycemia, ketoacidosis and micro-vascular complication (Sjöberg et al. 1987; Steffes et al. 2003; Madsbad et al. 1986; Lachin et al. 2014; Madsbad et al. 1979). C-peptide is used as a measure of endogenous insulin production and is tested either fasting or after mixed meal stimulation (Palmer et al. 2004; Besser et al. 2013; Garcia-Webb et al. 1982; Greenbaum et al. 2008).

Shortly after their diagnoses, most patients with T1D experience an increase in endogenous insulin release, which peaks approximately 3 months after onset. This is partly caused by beta-cell rest and by a reduction in the immune inflammatory conditions in the islets (Schloot et al. 2007; Aly and Gottlieb 2009). The remission or honeymoon phase is the period with sufficient endogenous insulin release to cover approximately 50 % of the insulin needed.

Many attempts have been made to prolong the remission phase through insulin prophylaxis to induce beta-cell rest, immune-modulating therapy such as anti-CD3 or anti-CD 20 or vaccines with GAD alumn, all without convincing effects (Ludvigsson 2007; Brown and Rother 2008; Keymeulen et al. 2010; Pescovitz et al. 2014; Ludvigsson et al. 2012). An alternative intervention is a gluten-free diet. This is motivated by animal studies where gluten has been shown to affect gut permeability, pro-inflammation, beta cells, natural killer (NK) cells and gut microbiota (Drago et al. 2006; Ejsing-Duun et al. 2008; Antvorskov et al. 2013; Dall et al. 2013; Marietta et al. 2013; Larsen et al. 2014).

Epidemiological evidence suggests that a gluten-free diet may have a positive effect in the protection against T1D in humans with coeliac disease. For instance, one study observed protection against the development of T1D in celiac patients adhering to gluten-free diet (Cosnes et al. 2008). Evidence, however, is conflicting as other studies found no protection (Viljamaa et al. 2005). The importance of gluten in T1D is highlighted by cohort studies finding that early introduction of gluten in the diet (before 3 months of age) increases the prevalence of diabetes autoantibodies fourfold in high-risk individuals (Norris et al. 2003; Ziegler et al. 2003). Two smaller studies have evaluated the effect of a 6-month or 1-year gluten-free diet in high-risk individuals before T1D with limited support of an effect on the level of autoantibodies and on the natural course towards diabetes (Hummel et al. 2002; Pastore et al. 2003; Fuchtenbusch et al. 2004). One of these studies did, however, show signs of improved insulin sensitivity as well as insulin secretion in the gluten-free diet group compared to the group on a normal diet (Pastore et al. 2003).

To our knowledge, the effect of a gluten-free diet at the onset of diabetes has not been systematically evaluated and, except for one case report (Sildorf et al. 2012), the effect of a gluten-free diet on the duration of PR and the SCP decline over time is unknown. The aims of the present study were therefore to test the feasibility of a gluten-free diet in children with newly diagnosed T1D as well as assessing the effect on diabetes outcome.

## Methods

This was an observational pilot study including15 children ranging from two to 15 years of age with newly diagnosed T1D admitted to the Paediatric Department at Copenhagen University Hospital, Herlev between March 2012 and June 2013. All health care providers were informed about the study. Exclusion criteria were children with non T1D, below 2 years of age, physical disabilities, psychiatric diagnosis, social or language problems. During this time period 73 patients with T1D above 2 years of age were admitted to Herlev with newly diagnosed diabetes, two came from another hospital because they had heard about the study. Of the 16 families included, one never started on a gluten-free diet and was excluded entirely. Another family dropped out after 6 months due to a loss of interest in continuing the gluten-free diet and in one child the boost test was not repeated because of immeasurable C-peptide after 6 months. In the remaining 13 children, who continued on the gluten-free diet, the adherence to the diet varied (Table 1).

**Table 1:** Age, gender autoantibody status at onset, SCP (pmol/l) and IDAA1C, as well as the parents’ answers regarding adherence to the gluten-free diet

Patients	Gender	Age at onset (year)	Autoantibodies ICA/GAD/IA2/IAA/ZnT8A p = positive n = negative	Adherence to gluten-free diet (%)	SCP at inclusion	SCP 6 months	SCP 12 months	IDAA1c 6 months	IDAA1C 12 months
1	F	2	p/p/p/p/p	90	57	<10	<10	9.29	–
2	M	3	p/n/p/p/p	85	269	–	52	8.97	8.63
3	F	3	p/n/p/p/n	100	–	244	109	6.99	10.4
4	M	5	p/n/p/p/p	100	566	308	86	5.99	7.81
5	M	6	p/p/p/p/p	100	384	303	214	6.92	7.86
6	M	8	p/n/p/n/p	–	520	427	153^a^	8.06	9.68
7	M	8	p/p/p/n/p	100	917	211	83	8.72	8.74
8	M	11	p/p/p/p/n	90	543	364	308	9.14	10.7
9	M	12	p/n/p/p/p	100	–	599	137	12.3	13.3
10	M	12	n/n/n/n/n	50	569	298	–	10.0	–
11	F	12	p/n/p/n/n	100	844	818	401	12.9	9.71
12	M	12	p/n/p/p/p	100	781	586^b^	682	7.03	6.95
13	M	13	p/n/p/p/p	50	503	530	298	8.66	8.97
14	M	13	p/p/p/n/p	100	1360	1770	1210	7.90	7.00
15	F	16	p/p/p/n/n	90	1240	904	245	7.89	11.8

The lowest value for adherence was chosen if the answers regarding adherence home and outside the home were different, in all cases the adherence outside home was lowest

^a^The pump was not stopped and there were 1.7 units of active insulin in the pump when boost was performed

^b^1 unit of insulin was given 1 h prior to boost

The local ethics committee (Region Hovedstadens Videnskabs etiske komité) approved the study (no. H-3-2011-119) and parents gave their written informed consent. Children with newly diagnosed T1D and their parents were invited to participate; those who accepted were given advice concerning a gluten-free diet by a dietician. They were monitored according to our normal routine except for a liquid mixed-meal challenge at inclusion and at their 6- and 12-month visits to stimulate endogenous C-peptide release. The intervention group was compared to two paediatric cohorts from European countries (1999–2000) and Denmark (2004–2005), including 378 children with new onset T1D described in detail elsewhere (Max Andersen et al. 2014a).

Adherence to the gluten-free diet was evaluated by a questionnaire, asking the child (if above 11 years of age) and the parents about their conception of the importance of a gluten-free diet (1–10 scale), as well as their adherence to the diet (‘How sure are you that the gluten-free diet is followed at home/outside the home’) (1–100 %).

The PR was defined based on measures of IDAA1c (insulin dose-adjusted A1c) and stimulated C-peptide. Patients were in PR if IDAA1c (4*insulin dose/kg/24 h + HbA1c in  %) ≤9 or C-peptide >300 pmol/L (Palmer et al. 2004; Mortensen et al. 2009; Max Andersen et al. 2014b).

In the gluten-free cohort, SCP was measured using two-site chemiluminescent immunometric assay (Immulite 2500) with a reportable range from 0.03 to 6.6 nmol/L and an efficient variance of 20 %. In the European cohort and in the Danish cohort the SCP was measured using Auto DELFIA C-peptide, PerkinElmer Life and Analytical Sciences Inc, Turku, Finland, with a sensitivity of 1 pmol/L and intraassay coefficient of variation below 6 %.

A liquid mixed-meal Boost™-test (6 mL/kg, max: 360 mL, Mead Johnson, Evansville, IN, USA; 237 mL = 9 FL OZ contains 33 g carbohydrate, 15 g protein, and 6 g fat, a total of 240 kcal) (Boost High Protein) was used to stimulate C-peptide in the European (1, 6 and 12 months after onset) and in the gluten-free diet study (inclusion, six and 12 months after onset); whereas Boost Original (237 mL containing 41 g carbohydrate, 10 g protein and 4 g fat, 249 kcal) was used to stimulate C-peptide in the Danish cohort (one, three, six and 12 months after onset). In all cohorts the test was in the morning after fasting for 8 h, the morning insulin dose being omitted. The SCP was measured 90 min after liquid mixed meals were ingested.

HbA1c was determined using a high-pressure liquid chromatographic method (Tosoh Bioscience, South San Francisco, CA, USA) working range 23.5–40 mmol/mol (4.3–5.8 %).

Blood glucose was measured as capillary values using Bayer contour at 0 and 90 min.

Carbohydrates and total energy intake were estimated during spring 2013 for all patients independent of the date of diagnosis using web-based Dietary Assessment Software for Children (WebDASC) developed and validated as an interactive food record recall method, as described in detail elsewhere (Biltoft-Jensen et al. 2013). The carbohydrate content in the diet was not a part of the protocol but chosen individually by the families. The results were compared with dietary intake among healthy children participating in the nationally representative survey of dietary habits (Pedersen et al. 2010).

## Statistics

Descriptive statistics and the non-parametric assessment of association based on Spearman’s rank correlation coefficient were used on dietary data. The numbers in PR were evaluated using logistic regression. Linear mixed models were used for the outcome parameters HbA1C, IDAA1c as well as log-transformed C-peptide, allowing for repeated measures in the same child by including a random child effect. The models included a fixed effect of cohort/intervention adjusted for diabetes duration and age. Goodness of fit was assessed graphically by means of residual plots and quantile plots. Only examination times with complete recordings of variables relevant to each of these analyses were included in the analyses.

All p values were evaluated at a 5 % significance level. Statistical analysis was performed using SAS version 9.3 (SAS Institute, Cary, NC, USA).

## Results

Patients diagnosed in the study period meeting inclusion criteria were invited to participate, only those expressing interest were approached for further information. Of those, approximately 80 % expressed interest in treatment prolonging remission, but less than half of the families were interested in intervention in the form of a gluten-free diet early after diagnosis.

Table 1 presents the age, gender and autoantibody status as well as the SCP and IDAA1c of the 15 patients in the study at inclusion. One patient was autoantibody negative for all autoantibodies (ICA, GADA, IA-2A, IAA, ZnT8A). The gender, mean age at onset, BMI z-score, and the corresponding diabetes duration (at inclusion) of the three cohorts are presented in Table 2, showing a significantly longer diabetes duration, higher SCP level and lower BMI at entry into the study in the gluten-free diet cohort.

**Table 2:** Unadjusted comparison of the three cohorts regarding gender, age, BMI z-score and SCP at inclusion, as well as the duration of diabetes when BMI and SCP were evaluated

Variable	Danish	European	Gluten-free	p value
Gender (M/F)	66/63	128/133	11/4	0.19
Age at onset in years (mean/SD)	10.0 (± 0.3)	9.1 (± 0.2)	9.1 (± 1.1)	0.06
z-BMI (mean/SD)	0.43 (0.1)	0.40 (± 0.1)	−0.1 (± 0.3)	0.30
C-peptide at inclusion pmol/L (median/range)	629 (10–1934)	409 (10–2040)	566 (57–1360)	<0.01
Duration at first visit in years (mean/SD)	0.09 (0.00)	0.10 (0.01)	0.21 (0.04)	<0.01

The differences in dietary intake show that the younger children (aged six to nine) obtained 29 % of their energy from carbohydrates, compared to 52 % in their apparently healthy counterparts from the national dietary survey. For the older group of children (aged 11–13) the energy contribution from carbohydrates was 43 %, compared to 53 % in their apparently healthy counterparts. There was no correlation between the percentage of energy from carbohydrates and HbA1c (r = 0.06; p = 0.87). There was a significant negative association between the daily exogenous insulin dose per kg and the percentage of energy from carbohydrates (r = −0.70; p = 0.02). Six out of 14 indicated adherence below 100 %, one never answered this question.

**Fig. 1:**
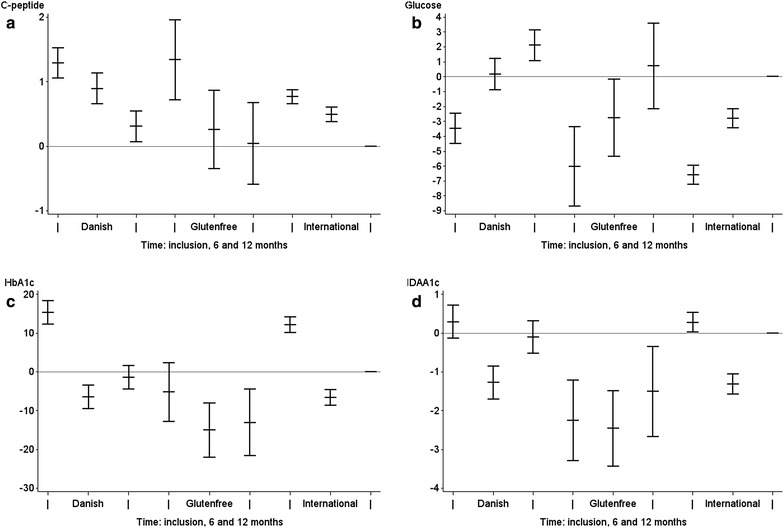
The differences between the gluten-free diet and the two previous cohorts at inclusion, after six months and 12 months. **a** Log(SCP) p = 0.0016 for differences between cohorts, and p = 0.36 for differences between intervention and the Danish cohort and p = 0.45 for the difference between intervention and European cohort. **b** Stimulated blood glucose; the test for overall difference was <0.0001, the p value for the difference between intervention and Danish cohort was p = 0.003, and for the difference between the intervention and European p = 0.81. **c** HbA1c (IFCC) in mmol/mol—the p < 0.01 for both the tested difference for Danish and intervention and European and intervention. **d** IDAA1c with similar results (p < 0.01)

HbA1c was significantly lower in the children on the gluten-free diet compared to both the Danish [−12.5 mmol/mol(CI 95 % −18.6; −6.3 mmol/mol)] and European cohorts [−12.3 mmol/mol (CI 95 % −18.3; −6.3 mmol/mol)] (p < 0.001), which corresponds to more than 20 % reduction in HbA1c. The same was found for IDAA1c, which was significantly lower in children on gluten-free diet than both the Danish-1.13 (CI 95 % −1.95; −0.32) and European cohorts −1.16 (CI 95 % −1.95; −0.37) (p < 0.01) (Fig. 1).

When log SCP was compared between the cohorts the estimated log (C-peptide) was 1.31 (CI 95 % 0.87; 1.99) (p = 0.20) times that of the gluten-free diet group in the Danish cohort; whereas the estimated log (C-peptide) in the European cohort was 0.88 (CI 95 % 0.59; 1.32) (p = 0.54) compared to the intervention group when adjusted for stimulated blood glucose. The Danish cohort was stimulated with a mixed meal containing more carbohydrate and less protein. The stimulated blood glucose was [2.7 mmol/L (CI 95 % 0.7; 4.7 mmol/L)] higher than the gluten-free (Fig. 1). The blood glucose was indifferent in the gluten-free diet cohort and the European cohort [−0.4 mmol/L (CI 95 % −2.4; 1.5 mmol/L)] where the same boost was used. The insulin dose per day was the same in all three cohorts (p > 0.05). The PR according to the different definitions is outlined in Table 3.

**Table 3:** PR after 6 and 12 months adjusted for BG stimulated

Variable	Danish	European	Gluten-free	p value G versus D	p value G versus I
*6 Months*					
PR IDAA1c (≤9)	56/118 (51.6 %)	104/235 (44.3 %)	11/15 (73.3 %)	0.51	0.01
PR SCP (>300 pmol/L)	83/117 (70.9 %)	114/237 (48.1 %)	10/14 (71.4 %)	0.55	0.08
*12 Months*					
PR IDAA1C (≤9)	23/119 (19.3 %)	41/233 (17.6 %)	7/13 (53.8 %)	0.02	<0.001
PR SCP (>300 pmol/L)	57/120 (47.5 %)	71/240 (29.6 %)	4/13 (30.8 %)	0.10	0.83

There was no significant difference according to injection method in the gluten-free cohort (p = 0.52), where those on insulin pumps had a mean HbA1c of 53.4 mmol/mol whereas, those on pens had a mean value of 51.4 mmol/mol.

After 6 months significantly more children were in PR defined by IDAA1c in the gluten-free diet cohort compared to the European cohort; whereas there was no difference between the gluten-free diet cohort and the Danish cohort. After 12 months three times as many children were in PR defined by IDAA1c in the gluten-free diet cohort compared to both the European and Danish cohort.

An intention to treat analysis was performed including data from the child who never started out on gluten-free diet and the overall results were the same—data not shown.

## Discussion

A gluten-free diet at diabetes onset is associated with improved HbA1c and three times as many subjects in PR (defined by IDAA1c) after 12 months. Families are interested in the gluten-free diet just after onset of T1D if the diet is likely to prolong remission and improve outcome. However, 100 % adherence to the diet is difficult to obtain.

To detect a 50 % reduction of the rate of decline in SCP, approximately 30 individuals are needed. Therefore, this study is underpowered to test a change of this magnitude. The overall tendency is a similar rate of decline in SCP in the three cohorts. Perhaps the results presented reflect improved insulin sensitivity in the gluten-free intervention. A gluten-free diet in pre-diabetes individuals led to a lower insulin resistance (HOMA-IR index), which increased when they returned to their normal diet (Pastore et al. 2003). If a gluten-free diet improves insulin sensitivity then the same amount of boost (6 ml/kg) during the observation period may give rise to lower postprandial glucose levels and stimulate beta cells less, thereby introducing a false lower C-peptide response.

The lower IDAA1c is due to the lower HbA1c since insulin dosing were the same, indicating the need for less insulin to reach the same HbA1c. Several beneficial effects of a gluten-free diet may explain the positive results regarding HbA1c and IDAA1c in the intervention group. Gluten (consisting of gliadin and glutenins) in the diet is known to increase gut permeability and T1D patients have increased gut permeability in comparison to healthy subjects (Bosi et al. 2006). Increased permeability allows macromolecules to pass from the gut to the bloodstream and may interfere with the inflammatory system, e.g. change the Th1/Th2 balance towards a more anti-inflammatory immune reaction and increase NK-cell activity (Drago et al. 2006; Ejsing-Duun et al. 2008; Antvorskov et al. 2013; Larsen et al. 2014). Besides this, gluten-free diet changes the intestinal flora (Marietta et al. 2013). Of special interest is the fact that Akkermansia mucinophilic bacteria increase in number. They eat the mucus layer in the intestine, giving rise to increased mucin synthesis and tight junctions, leading to better gut integrity. They have also been associated with diabetes protection (Hansen et al. 2012). Recently it has been shown that uptake of/absorbed gliadin fragments are transported to the pancreas, where they increase insulin secretion and thereby exhaust the beta cells (Dall et al. 2013).

The low carbohydrate intake in the intervention group in comparison to healthy school children and probably the two previous cohorts could explain lower IDAA1c levels (Arora and McFarlane 2005; Rovner et al. 2009). Though, we could not confirm an association between carbohydrate in the diet and HbA1c, and the association between insulin dose and carbohydrate in the diet is the opposite of what was expected. These results speak against explaining the difference in IDDA1c by carbohydrate content. In general, children with diabetes tend to eat healthier than the general population (Due et al. 2013). The gluten-free cohort had lower HbA1c and relatively higher SCP at inclusion; this could be due to the timing of first C-peptide measure since SCP after onset seems to peak around 3 months after onset. Another limitation is the cohorts represent different treatment periods. This could, however, lead to an under-estimated effect of gluten-free diet as individuals studied more recently had a faster rate of decline of C-peptide over time when compared to older cohorts of children with T1D (Max Andersen et al. 2014a). None in the two previous cohorts were using insulin pumps. In the older cohorts there may be factors influencing the insulin dosing such as use of insulin analogs and treatment target may have changed. On the other hand, the HbA1c levels was similar in those on pumps and pens, indicating that type of injection method could not explain the better HbA1c. Furthermore, HbA1c levels has not decreased significantly in the Danish population from 2005 until 2012 despite increased use of intensive treatment (Sildorf et al. 2016).

The strength of this study is the comprehensive investigation of these patients, including information on diet adherence and diet composition. The results call for further research into a gluten-free diet, since a gluten-free diet has no adverse effects except for the inconvenience associated with avoiding gluten. Even if the effect is modest, studies show that even a limited amount of endogenous C-peptide improves acute and long-term complications (Steffes et al. 2003; Lachin et al. 2014). In two cases insulin was given prior to boost test which may have led to a lower level of SCP and subsequent under-estimation of the effect of gluten-free diet. Additional it is a drawback that assays for C-peptide measures may have changed from the historical cohorts. Other limitations of the study include the small sample size and the non-randomised design.

Intervention studies in children that are aimed to preserve beta-cell function are important since children are more likely to experience a beneficial effect due to a higher degree of beta-cell replication compared to adults (Meier et al. 2008). Furthermore, diet intervention is a multifactorial approach because gluten may affect not only the immune system and the gut microbiota, but may also have local effects in the pancreas. An advantage of intervention at onset is that there is no over-treatment of children not at risk of T1D.

The increased burden placed on the families by introducing an extra dietary intervention beside the diabetes diet has to be balanced, since even psychological stress has been shown to affect the immune system (Carlsson et al. 2014). Any attempts to start children on a gluten-free diet at disease onset should be accompanied by a discussion of the extra burden and a careful evaluation of the family’s ability to cope with the diet without stress. On the other hand, some families may experience positive feelings when they actively do something to fight the C-peptide decline.

## Conclusion

Gluten-free diet is feasible and, in this study, shows better metabolic control as assessed by HbA1c and IDAA1c and three times higher prevalence of PR 1 year after diagnosis, which is likely to improve long-term outcome (Cleary et al. 2001; Olsen et al. 2004). This results needs further confirmation as it is based on a with older cohorts with a less pronounced rate of decline in SCP. The true effect of a gluten-free diet on the immune system in humans and the natural course of SCP decline needs to be addressed in a randomized control trial.

## Abbreviations

T1D: type 1 diabetes
IDAA1c: insulin dose-adjusted HbA1c
PR: partial remission
SCP: stimulated C-peptide
